# Assessment of Childbirth-Related PTSD: Psychometric Properties of the German Version of the City Birth Trauma Scale

**DOI:** 10.3389/fpsyt.2021.731537

**Published:** 2021-10-06

**Authors:** Tobias Weigl, Franziska Marie Lea Beck-Hiestermann, Nikola Maria Stenzel, Sven Benson, Manfred Schedlowski, Susan Garthus-Niegel

**Affiliations:** ^1^Psychology School, Hochschule Fresenius - University of Applied Sciences Düsseldorf, Düsseldorf, Germany; ^2^Department of Clinical Psychology and Psychotherapy, Psychologische Hochschule Berlin, Berlin, Germany; ^3^Institute of Medical Psychology and Behavioral Immunobiology, Center for Translational Neuro- and Behavioral Sciences, University Hospital Essen, University Duisburg-Essen, Essen, Germany; ^4^Department of Clinical Neuroscience, Osher Center for Integrative Medicine, Karolinska Institutet, Stockholm, Sweden; ^5^Institute for Systems Medicine (ISM), Faculty of Human Medicine, Medical School Hamburg, Hamburg, Germany; ^6^Department of Child Health and Development, Norwegian Institute of Public Health, Oslo, Norway; ^7^Faculty of Medicine, Institute and Policlinic of Occupational and Social Medicine, Technische Universität Dresden, Dresden, Germany

**Keywords:** posttraumatic stress disorder, childbirth, mothers, perinatal mental health, questionnaire, DSM-5

## Abstract

**Background:** About 3–4% of women in community samples suffer from childbirth-related posttraumatic stress disorder (PTSD). Surprisingly, the recently developed City Birth Trauma Scale (City BiTS) was the first diagnostic tool for childbirth-related PTSD covering DSM-5 criteria for PTSD. Since no questionnaire on childbirth-related PTSD is available in German, we aimed to validate a German translation of the City BiTS and to provide information on its psychometric properties.

**Methods:** A community sample of 1,072 mothers completed an online survey, which included questions on sociodemographic and obstetric characteristics, the German version of the City BiTS, the Impact of Event Scale-Revised (IES-R), the PTSD Checklist for DSM-5 (PCL-5), Edinburgh Postnatal Depression Scale (EPDS), and the anxiety subscale of the Depression, Anxiety, and Stress Scale (DASS-Anxiety).

**Results:** Exploratory factor analysis (EFA) on a random split-half sample confirmed the previously reported two-factorial structure of the City BiTS. The factors “Childbirth-related symptoms” and “General symptoms” explained about 53%, 52% of variance. Internal consistency was good to excellent for the subscales and the total scale (Cronbach's Alpha = 0.89−0.92). In a confirmatory factor analysis (CFA) in the holdout sample the two-factorial solution reached the best model fit out of three models. Correlation analyses showed convergent validity of the City BiTS (total scale and subscales) with the IES-R and PCL-5 and divergent validity with the EPDS and the DASS-Anxiety.

**Limitations:** Data were acquired in a community sample and prevalence rates might not be representative for mothers of high-risk groups, e.g., after preterm birth.

**Conclusions:** The German version of the City BiTS is the first German questionnaire which allows to assess symptoms of childbirth-related PTSD according to DSM-5 criteria. Besides an improvement in clinical routine it will help to make data on prevalence of childbirth-related PTSD internationally comparable. In addition, this work provides a basis to assess childbirth-related PTSD in studies conducted with a longitudinal study design or in high-risk samples.

## Introduction

Recent data suggest, that giving birth even without any further medical complications for mother or child may lead to PTSD in 3–4% of women and the prevalence might increase during the first year postpartum (1–4). In populations with risk factors such as operative birth or giving birth to a child with very low birth weight, even up to 15.7 or 18.5% of women might be affected (3, 4). The Diagnostic and Statistical Manual of Mental Disorders (DSM-5) refers to posttraumatic stress disorder (PTSD) as a reaction to events in which a person is exposed to “actual or threatened death or serious injury, or a threat to the physical integrity of self or others” (5). Symptoms include intrusions, avoidance of trauma-related stimuli, alterations in mood and cognitions, as well as alterations in arousal and reactivity (5). While accidents, wars, or natural disasters represent well-known traumatic stressors, childbirth may also meet DSM criteria for a traumatic event (6). Yet, childbirth-related PTSD is not regularly assessed in postpartum routine care.

Adverse effects of childbirth-related PTSD are not limited to impaired maternal mental health. In fact, consequences may include a negative impact on the couple relationship, the parent-child relationship, or even the child's development (7–12). Therefore, early identification of childbirth-related PTSD may be beneficial for the whole family. Self-report questionnaires offer an economic and efficient way for the assessment of mental health problems in the peripartum period (13). So far, three questionnaires have been developed to assess symptoms of childbirth-related PTSD. Two of them however, the Traumatic Event Scale as well as the Perinatal PTSD Questionnaire, are based on outdated diagnostic criteria from DSM-IV (14–16). Only the recently developed City Birth Trauma Scale (City BiTS) was established in consideration of diagnostic criteria from DSM-5 (17). Next to the original English version, the City BiTS has been validated and translated into Turkish, Spanish, Hebrew, Croatian, and French (18–22). In addition to identifying the need for individual prevention and treatment of childbirth-related PTSD, cross-cultural validations will help to establish the City BiTS as a standardized measure internationally, allowing for comparisons of childbirth-related PTSD symptoms between different languages and cultures.

To date there exists no validated German measure to assess childbirth-related PTSD (23). This is a hindrance in research and clinical care. To close this gap, the aim of the present study was therefore to provide a translated German version of the City BiTS and to determine its psychometric properties in a community sample.

## Materials and Methods

### Study Population and Design

The cross-sectional data presented in this article are part of the longitudinal “LABOR”-study (Longitudinal Analysis of Birth mode and Outcomes Related) in which three different groups of women were recruited: pregnant women, women who had their baby within the last 12 months, and women who had their baby more than 12 months ago. After their first assessment, all participants were asked if they were willing to take part in further follow-up assessments, scheduled at 3, 6, and 9 months after study entry. For the present study, only women of the second group, i.e., women who gave birth within the last 12 months were included. Exclusion criteria were death of the child during or after birth, younger age than 18 years, and insufficient German language skills. Invitations to take part in the study were posted on social media, such as Instagram and Facebook, as well as parents' blogs and forums between February and April 2020. The study was conducted online with the use of the platform EFS survey by QuestBack (unipark.de). Several arrangements ensured high quality of data. The platform offers the possibility to block participants from completing the questionnaire more than once. To ensure that participation was not only motivated by incentives like money or gift cards, women did not receive any kind of compensation. Since answers were obligatory for the participants, no imputation for missing values had to be conducted. Participants could complete the questionnaire on a smartphone or computer with an active internet connection in an environment of their choice. Participants were informed that some of the questions might relate to unpleasant or even traumatic experiences, which might trigger unwanted memories and emotions. Further, participants were informed about their right to withdraw from the study at any given time and were advised to seek professional help if needed (informed consent included a list of mental health services like the German National Suicide and Crisis Line). A total of 1,072 women who met inclusion criteria gave informed consent to follow the study protocol. Ethical approval was granted by the ethics committee of the Psychologische Hochschule Berlin (approval no. AZ: EK201921-II) and the study was performed in accordance with the Declaration of Helsinki. All data were stored anonymously and in accordance with the German General Data Protection Regulation (GDPR). On average, it took participants around 45 min to complete the whole assessment, including further questionnaires, which were not relevant for this study. Participants could pause and continue the questionnaire afterwards.

### Measures

#### Sociodemographic and Obstetric Characteristics

Sociodemographic information was assessed including maternal age, relationship status, as well as educational status. Questions regarding obstetric characteristics included length of gestation, infant age, parity, type of delivery [spontaneous vaginal delivery, instrumental vaginal delivery, emergency, or planned cesarean section (CS)], pregnancy at risk (yes/no), and preterm birth (see Table 1 for further details).

**Table 1:** Sociodemographic and obstetric characteristics of the sample.

**Variables**	**(*N* = 1,072)**
*Age of mothers (years)^a^*	30.6 ± 4.7
	(18–44)
*Relationship status (n, %)*	
Engaged/Married	808, 75.4
Cohabitating	240, 22.4
Divorced/Living apart	24, 2.2
*Education (n, %)*	
No degree	1, 0.1
Lower secondary education level 2/Secondary school certificate	172, 16.0
University entrance qualification	360, 33.6
University degree	539, 50.3
*German citizenship (n, %)*	1,003, 93.7
*Length of gestation (weeks)^a^*	39.7 ± 1.9
	(26–42)
*Infant age (days)^a^*	185.7 ± 101.1
	(5–366)
*Type of delivery (n, %)*	
Spontaneous vaginal delivery	739, 68.9
Instrumental vaginal delivery	90, 8.4
Planned CS	75, 7.0
Emergency CS	168, 15.7
*Primipara (n, %)*	653, 60.9
*Pregnancy at risk (n, %)*	246, 22.9
*Preterm birth (≤ 37 weeks; n, %)*	54, 5.0

a *M ± SD (range)*.

#### City Birth Trauma Scale

The City BiTS is a self-report questionnaire with 29 items, which was developed based on DSM-5 criteria in order to assess childbirth-related PTSD in women (17). Two dichotomous items (yes/no) assess the stressor criterion (i.e., threatened death or serious injury of the mother or the baby during labor, birth, or immediately afterwards). The frequency of intrusion, avoidance, negative cognitions/mood, and hyperarousal symptoms in the week prior to assessment is measured with 20 items. Items are rated on a four-point Likert scale from 0 (never) to 3 (5 or more times) to produce a sum score ranging from 0 to 60, with higher scores indicating elevated levels of PTSD-symptoms. Two additional items allow to screen for a dissociative subtype of PTSD. Another five items assess the onset of symptoms (from before the birth to more than 6 months after birth), duration of symptoms (from <1 month to >3 months), distress (yes/sometimes/no) as well as impairment (yes/sometimes/no), and exclusion criteria (i.e., symptoms due to medication, alcohol, drugs, or physical illness; yes/maybe/no).

In the original validation study, the subscales “Birth-related symptoms” (predominantly consisting of items which measure intrusion, avoidance, and negative cognitions/mood related to birth) and “General symptoms” (mainly consisting of items assessing negative cognitions/mood and hyperarousal) have been identified. The original version of the City BiTS showed excellent reliability with Cronbach's Alpha = 0.92 for the total scale (17). Upon consent from the original author (Susan Ayers), the City BiTS was translated into German, using the back-translation method (24). The back-translation was then discussed with the original author. This resulted in minor adjustments in the wording. Additionally, 10 participants of a pilot sample were asked to complete the German version of the City BiTS and to express any difficulties they had regarding comprehensibility. This did not result in any changes of the questionnaire.

#### Impact of Event Scale-Revised

The Impact of Event Scale-Revised (IES-R) is a self-report questionnaire assessing symptoms of PTSD in accordance with criteria of the DSM-IV (25). After specifying a certain stressful life event, items can be answered on a four-point scale with the response options 0 (not at all), 1 (rarely), 3 (sometimes), and 5 (often) referring to the last 7 days. In our study, women were instructed to refer to their birth experience only. Scores can be calculated for the Intrusion (7 items), Avoidance (8 items), and Hyperarousal (7 items) subscales. For the German version, authors advise against a total sum score and suggest an algorithm, which has been used in this study. Values above zero indicate probable PTSD (26).

#### PTSD Checklist for DSM-5

Symptoms of PTSD were also assessed with the PTSD Checklist for DSM-5 (PCL-5). The PCL-5 is a self-report measure comprising 20 items based on the DSM-5, and can be subdivided in the clusters intrusion (items 1–5), avoidance (items 6–7), negative mood and cognition (items 8–14), and hyperarousal (items 15–20). Each item reflects the severity of a PTSD symptom during the month prior to assessment. Ratings are carried out on a five-point Likert scale from 0 (not at all) to 4 (extremely). The German version was used and women were instructed to answer in relation to the birth of their last child (27).

#### Edinburgh Postnatal Depression Scale

Symptoms of depression were measured with the German version of the Edinburgh Postnatal Depression Scale (EPDS), the most common self-report scale to assess depression in the postpartum period (28, 29). The EPDS consists of 10 items. With a four-point scale from 0 to 3, the sum score ranges from 0 to 30. Higher scores reflect higher levels of depression. A cut-off ≥10 indicates a substantial level of depressive symptoms suggesting that further diagnostic procedures should be performed (29, 30).

#### Depression, Anxiety, and Stress Scale

The Depression, Anxiety, and Stress Scale-21 (DASS-21) consists of 21 items with three subscales assessing symptoms of depression, anxiety, and stress and has been validated in postpartum mothers (31, 32). Items can be rated on a scale from 0 (did not apply to me at all) to 3 (applies to me very much or most of the time), and sum scores can be calculated for each scale. In the present study, the German version of the subscale “DASS-Anxiety” was used (33).

### Statistical Analysis

Exploratory factor analysis (EFA) on a random split-half sample (*n* = 536), calculations of correlation coefficients for convergent and divergent validity, and tests for group differences were performed using IBM SPSS statistics version 27 for windows. Univariate ANOVA with Bonferroni *post-hoc* comparisons were used to analyze potential differences regarding type of delivery. Confirmatory factor analysis (CFA) in the holdout sample (*n* = 536) was performed using IBM SPSS Amos version 27. Fit indices included Root Mean Square Error of Approximation (RMSEA), Standardized Root Mean Square Residual (SRMR), Comparative Fit Index (CFI), and Tucker-Lewis Index (TLI). Good (and adequate, respectively) model fit is indicated by RMSEA ≤ 0.06 (0.06–0.08), SRMR ≤ 0.08, and CFI as well as TLI ≥0.95 (0.90–0.95) (34).

## Results

### Sample Characteristics

A total of *N* = 1,072 mothers were included in the final sample (age: *M* = 30.6, *SD* = 4.7). Almost all women were in a permanent relationship. Most children were born by spontaneous vaginal birth (*n* = 739) and for approximately 60% of mothers it was their first birth. Only 5% of births took place before gestational week 37 (see Table 1 for further details).

Responses to the City BiTS in accordance with DSM-5 criteria for PTSD are shown in Table 2. About 22% women fulfilled the stressor criterion by indicating that they believed they or their baby would be seriously injured or even die during childbirth (or immediately afterwards). A total of 28 (2.6%) women fulfilled all criteria to qualify for a PTSD diagnosis according to DSM-5 criteria.

**Table 2:** Assessment of PTSD with the City BiTS using DSM-5 diagnostic criteria (*N* = 1,072).

**Criterion**		***n*, %**
A	Stressor criterion (“yes-response” on item 1 or item 2)	232, 21.6
B	Intrusion symptoms (1 needed)	519, 48.4
C	Avoidance symptoms (1 needed)	199, 18.6
D	Negative cognitions and mood (2 needed)	517, 48.2
E	Hyperarousal (2 needed)	637, 59.4
F	Duration (more than 1 month necessary)	538, 50.2
G	Distress and impairment (“yes-response” on item 27 or item 28)	157, 14.6
H	Exclusion criteria (“yes-response” on item 29)	13, 1.2
	Diagnostic criteria met for PTSD	28, 2.6

### Exploratory Factor Analysis

With a Kaiser-Meyer-Olkin coefficient of 0.92 and a chi-squared value of (χ^2^ = 5336.06; *df* = 190; *p* < 0.001) in the Bartlett's test of sphericity, results indicated that the sample can be used for factor analysis (35, 36).

In accordance with previous validations of the City BiTS, an EFA using principal component analysis with varimax rotation was conducted (18, 21). Applying Kaiser's rule (retain Eigenvalues >1) and the screen test, a structure with two factors could be found. Factor 1 included items 3–12 and factor 2 included items 13–22. Items 23 and 24 were excluded from EFA, since they can be used to measure dissociative symptoms but do not represent main symptoms of PTSD. In our study, the total scale accounted for almost 52% of variance, with the subscales “Birth-related symptoms” and “General symptoms” explaining roughly 38 and 14% of the variance, respectively (see Table 3 for further details).

**Table 3:** Factor loadings of the EFA with two factors in principal component analysis with varimax rotation.

**Item**		**Birth-related symptoms**	**General symptoms**
**B**	**Intrusions**		
3	Recurrent unwanted memories of the birth (or parts of the birth) that you can't control	**0.789**	0.149
4	Bad dreams or nightmares about the birth (or related to the birth)	**0.565**	0.114
5	Flashbacks to the birth and/or reliving the experience	**0.588**	0.147
6	Getting upset when reminded of the birth	**0.806**	0.157
7	Feeling tense or anxious when reminded of the birth	**0.784**	0.232
**C**	**Avoidance**		
8	Trying to avoid thinking about the birth	**0.715**	0.203
9	Trying to avoid things that remind me of the birth (e.g., people, places, TV programs)	**0.620**	0.221
**D**	**Negative mood and cognitions**		
10	Not being able to remember details of the birth	**0.455**	0.063
11	Blaming myself or others for what happened during the birth	**0.752**	0.168
12	Feeling strong negative emotions about the birth (e.g., fear, anger, shame)	**0.793**	0.246
13	Feeling negative about myself or thinking something awful will happen	0.277	**0.687**
14	Lost interest in activities that were important to me	0.119	**0.739**
15	Feeling detached from other people	0.154	**0.735**
16	Not able to feel positive emotions (e.g., happy, excited)	0.144	**0.705**
**E**	**Hyperarousal**		
17	Feeling irritable or aggressive	0.110	**0.770**
18	Feeling self-destructive or acting recklessly	0.068	**0.486**
19	Feeling tense and on edge	0.191	**0.773**
20	Feeling jumpy or easily startled	0.250	**0.702**
21	Problems concentrating	0.211	**0.615**
22	Not sleeping well-because of things that aren't due to the baby's sleep pattern	0.201	**0.661**
	Percentage of variance explained by the factors	37.8	13.7

*The bold values represent the highest loading of items on each of the factors*.

### Confirmatory Factor Analysis

Based on theoretical reasoning and results of previous studies, the fit of three different models was tested in CFA. Firstly, we tested a model with four factors as determined in the diagnostic criteria of PTSD in the DSM-5, including the dimensions Intrusions (items 3–7), Avoidance (items 8 and 9), Negative cognitions/mood (items 10–16), and Hyperarousal (items 17–20). This analysis showed a poor fit of the model with four factors [$\chi_{\left(164\right)}^{2}$ = 1288.86, χ^2^/*df* = 7.86, RMSEA = 0.113, SRMR = 0.093, CFI = 0.81, TLI = 0.78].

Secondly, we tested a model with one overall factor. This model also had to be rejected [$\chi_{\left(170\right)}^{2}$ = 2050.71, χ^2^/*df* = 12.06, RMSEA = 0.144, SRMR = 0.119, CFI = 0.68, TLI = 0.64].

Thirdly, we tested a model with two factors that has previously been shown to yield an acceptable to good model fit. However, in one prior study minor modifications of the model (i.e., deletion of item 8) were necessary to reach acceptable model fit (18). In the current study, the two-factor model with two correlated dimensions in accordance with the original validation (“Birth-related symptoms” and “General symptoms”) was the best fit to the data according to all fit indices [$\chi_{\left(169\right)}^{2}$ = 771.27, χ^2^/*df* = 4.56, RMSEA = 0.082, SRMR = 0.056, CFI = 0.90, TLI = 0.89]. Yet, the 90% confidence interval for the RMSEA (0.076−0.088) was slightly above the suggested cut-off of 0.08 (37). However, since items of the City BiTS aim to measure symptoms of childbirth-related PTSD according to DSM-5 we decided against deletion of items.

### Reliability

Analysis of the internal consistency by Cronbach's Alpha resulted in good to excellent reliability of 0.90 for the “Birth-related symptoms” subscale, 0.89 for the “General symptoms” subscale, as well as 0.92 for the total scale. Deletion of any of the items did not increase Cronbach's Alpha substantially.

### Convergent and Divergent Validity

Convergent and divergent validity were tested by correlating both subscales and the total scale of the City BiTS against the IES-R, PCL-5, EPDS, and the DASS-Anxiety subscale (see Table 4).

**Table 4:** Intercorrelations of the City BiTS (subscales and total scale) with measures of PTSD symptomatology, depression, and anxiety (*N* = 1,072).

	***M (SD)***	**1**.	**2**.	**3**.	**4**.	**5**.	**6**.	**7**.
1. City BiTS: Birth-related symptoms	4.07 (6.05)	–	0.504^**^	0.857^**^	0.768^**^	0.789^**^	0.417^**^	0.448^**^
2. City BiTS: General symptoms	6.10 (6.49)		–	0.877^**^	0.635^**^	0.734^**^	0.688^**^	0.586^**^
3. City BiTS: Total scale	10.16 (10.88)			–	0.806^**^	0.877^**^	0.642^**^	0.598^**^
4. IES-R	−3.53 (1.37)				–	0.829^**^	0.515^**^	0.588^**^
5. PCL-5	9.65 (11.29)					–	0.653^**^	0.627^**^
6. EPDS	7.33 (5.49)						–	0.592^**^
7. DASS—Anxiety	1.83 (2.79)							–

** *p < 0.01*.

### Group Comparisons for Type of Delivery

**Figure 1:**
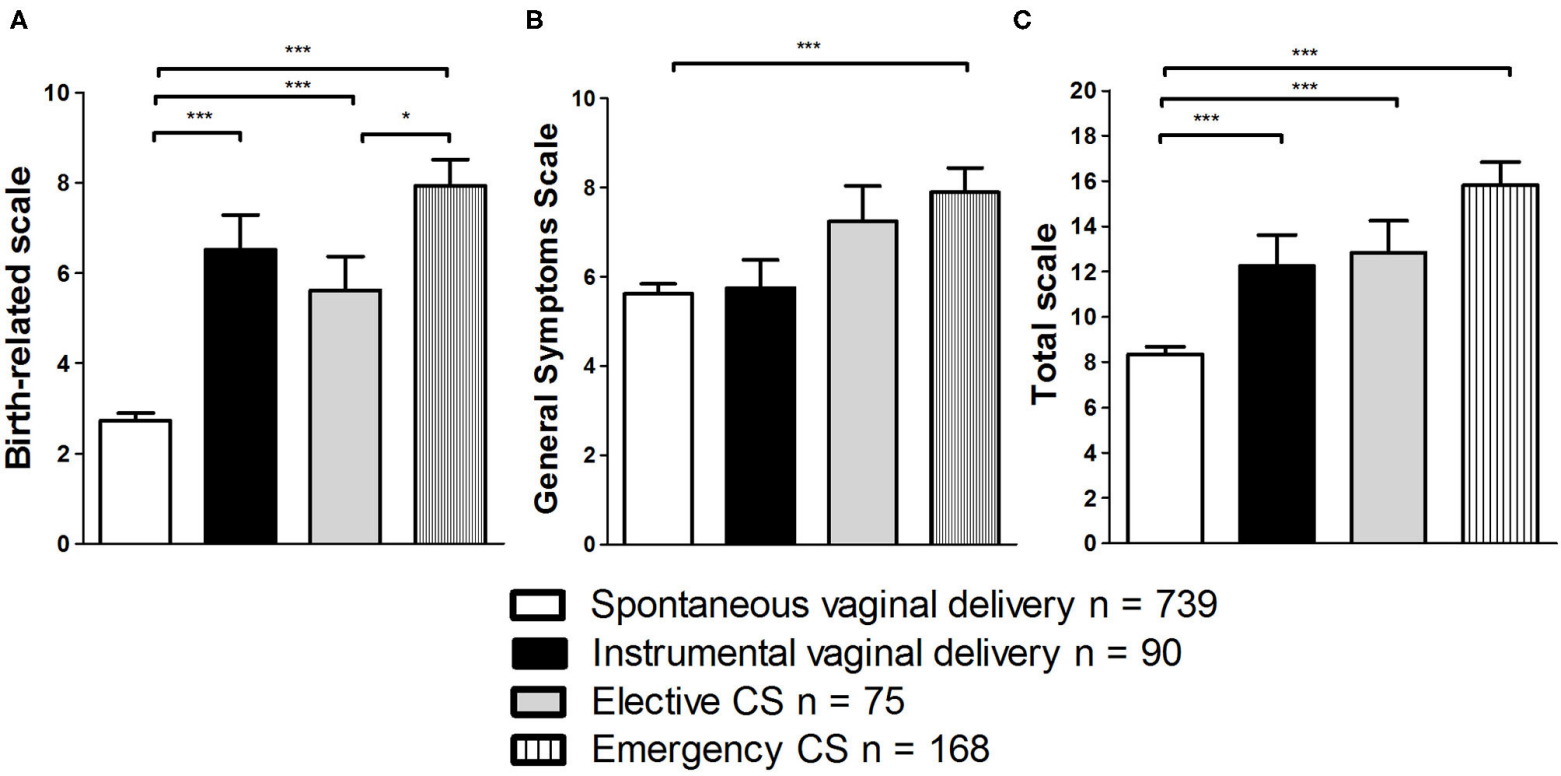
Comparison of effects of type of delivery on scores in City BiTS subscales “Birth-related symptoms” **(A)**, “General symptoms” **(B)**, and the total scale **(C)**. *p < 0.05; ***p < 0.001. Data are shown as mean values ± SEM.

Univariate ANOVA was employed to assess if more invasive and medically complicated types of delivery lead to more severe symptoms of PTSD. Main effects could be found for the subscales “Birth-related symptoms” and “General symptoms” as well as the total scale (all *p* < 0.001; see Table 5; Figure 1). Bonferroni *post-hoc* comparisons revealed that for the subscale “Birth-related symptoms” scores differed statistically significantly between spontaneous vaginal birth and all other types of delivery (all *p* < 0.001). Furthermore, there was a statistically significant difference between elective and emergency CS (*p* = 0.02). The only statistically significant difference found for scores of the scale “General symptoms” was between the groups spontaneous vaginal delivery and emergency CS (*p* < 0.001). For the score of the total scale the *post-hoc* comparisons revealed that the group spontaneous vaginal delivery differed from all other groups (*p* < 0.01). Other group differences could not be found (all *n.s*.).

**Table 5:** Differences in the City BiTS subscales and the total scale for type of delivery.

	**Birth-related symptoms**	**General symptoms**	**Total scale**
**Type of delivery**	***M* (*SD*)**	***M* (*SD*)**	***M* (*SD*)**
Spontaneous vaginal delivery (*n* = 739)	2.73 (4.82)	5.61 (6.27)	8.35 (9.28)
Instrumental vaginal delivery (*n* = 90)	6.51 (7.39)	5.74 (6.03)	12.26 (12.91)
Elective CS (*n* = 75)	5.61 (6.57)	7.24 (6.92)	12.85 (12.11)
Emergency CS (*n* = 168)	7.94 (7.53)	7.89 (7.15)	15.83 (13.15)
	*F*_(3, 1068)_ = 46.75; *p* <0.001	*F*_(3, 1068)_ = 6.58; *p* <0.001	*F*_(3, 1068)_ = 26.42; *p* <0.001

## Discussion

This study set out to provide a translated German version of the City BiTS and to determine its psychometric properties resulting in the first questionnaire to assess childbirth-related PTSD based on DSM-5 criteria in German language. The results of our study provide further important knowledge on the psychometric properties of the City BiTS. Also for the German version a two-factorial solution could be found in EFA, which is in accordance with previous validation studies, both of the original English version as well as other translated versions (17–22). The subscale “Birth-related symptoms” explained about 38% and the subscale “General symptoms” about 14% of variance. Both subscales as well as the total scale showed good to excellent internal consistency in the tested sample. Furthermore, using CFA we tested the model fit for three different models. Neither a four-factorial model based on diagnostic criteria of PTSD in DSM-5, nor a one-factorial solution achieved adequate fit. The best model fit was achieved for the two-factorial structure, which is comparable to previous studies (18, 20, 21). Still, when assessing the model fit of this two-factor model, results of the CFA showed that some of the fit indices were only close to suggested cut-offs (34, 37). However, methodological literature in the field suggests that in applied research universal cut-off criteria recommended for CFA might be too strict and therefore arguable (38–42). In fact, these cut-offs were derived from simulation studies and are hard to be replicated. Thus, cut-offs in CFA should not be overgeneralized (34, 40). Given that there is a clear theoretical rationale underlying the two-factorial solution, the combination of fit metrics in our study can be interpreted as an acceptable fit of the model. Since the composition of samples can affect results of a CFA, we suggest further validations of the German version of the City BiTS also in different samples and subsequent comparisons in relation to model fit.

By using the IES-R, the PCL-5, the EPDS, and the DASS-Anxiety in our study, the instruments to examine convergent and divergent validity were similar to previous studies (19, 20). Since the PCL-5 is based on the DSM-5, it offered additional information on the convergent validity of the City BiTS. Regarding convergent and divergent validity, results need to be differentiated. The correlation between scores of depression and anxiety with the subscale “Birth-related symptoms” was weaker than with the subscale “General symptoms” and the total scale of the City BiTS, confirming divergent validity. As to be expected, all scales of the City BiTS showed moderate to strong correlations with other measures of PTSD. Thus, convergent validity could be confirmed.

Additionally, group comparisons could show that the subscale “Birth-related symptoms” differentiates well-between types of delivery. Spontaneous vaginal delivery seems to result in only few childbirth-related symptoms. In contrast, emergency CS led to highest maternal symptom load, in both subscales as well as in the total scale. These results once more confirm, that even though all mothers may potentially suffer from childbirth-related symptoms of PTSD, women with deliveries other than a spontaneous vaginal delivery are at higher risk to develop such symptoms. Interventions should take this fact into account (43). To date, a cut-off for the City BiTS has not yet been established. Clinical interviews can be considered the gold standard for the assessment of mental disorders and should be applied as an external criterion to establish a cut-off, which might help to use the City BiTS in perinatal healthcare services more easily. Nonetheless, even in the current form the City BiTS represents an efficient and economic instrument and may play an important role in the prevention and intervention of childbirth-related PTSD. Once women suffering from symptoms of childbirth-related PTSD are detected, they can be assigned to psychotherapy. Promising and viable techniques are cognitive behavioral therapy, eye movement desensitization and reprocessing, as well as debriefing (44). Besides, the multitude of translations of the City BiTS are vital for global research on childbirth-related PTSD. Initiatives like the International Survey of Childbirth-Related Trauma (INTERSECT) are therefore able to study prevalence's of childbirth-related PTSD globally and make results directly comparable (45).

### Strengths and Limitations

The study has noteworthy strengths such as the large sample size and the use of several different measures to establish convergent and divergent validity. Most notably, our translation of the City BiTS is the first German questionnaire, which offers the possibility to assess childbirth-related PTSD on the basis of DSM-5 diagnostic criteria. Therefore, standardized use of the German version of the City BiTS in postpartum women could improve perinatal healthcare fundamentally. Yet, there are also limitations that need to be acknowledged. Our results were obtained from cross-sectional data and preexisting symptoms of mental disorders were not assessed. Thus, we cannot determine whether women who already suffered from symptoms of PTSD or other mental disorders exhibit different scores on the City BiTS than women with no such history. Therefore, future research should apply longitudinal designs measuring symptoms of childbirth-related PTSD at several points in time to allow for an estimation of trajectories. This could facilitate early identification of women at risk to suffer from chronic childbirth-related symptoms of PTSD (4, 46–48). Further, even though the sample size was large, a selection bias cannot be ruled out. Web-based surveys offer easy accessibility, but women who use social media on a regular basis are more likely to be represented in online samples (49). Thus, further studies should aim to recruit more representative samples of women. Additionally, in our sample only a low percentage of women fulfilled DSM-5 diagnostic criteria for PTSD. Even though the results are similar to previous studies, subsequent validation studies should include populations at a comparably higher risk for childbirth-related PTSD (3, 4, 50). Additional efforts should be made to examine women belonging to different ethnic groups.

### Conclusions

The findings of the present study provide strong evidence that the German version of the City BiTS offers adequate psychometric properties. This version is fit for use and perinatal healthcare might improve by its application in postpartum women. Furthermore, a questionnaire especially developed to assess childbirth-related PTSD will hopefully heighten the acceptance of standardized measurement of mental health in the peripartum period. Due to its explicit wording, women might relate more to a questionnaire on childbirth-related PTSD than to more general questionnaires assessing PTSD which might make their participation in postpartum psychological assessment more likely. As a result, childbirth-related PTSD might get more widely recognized by healthcare providers and affected women. In addition, efficient identification of women suffering from symptoms of childbirth-related PTSD will expedite allocation to adequate treatment for PTSD and might prevent them from being treated for e.g., postpartum depression instead. Based on this knowledge, evidence-based prevention and intervention strategies for childbirth-related PTSD can be refined in the future. In summary, the German version of the City BiTS may represent a useful tool in clinical routine and could help to make prevalence estimates of childbirth-related PTSD comparable across different countries.

## Data Availability Statement

The raw data supporting the conclusions of this article will be made available by the authors, without undue reservation.

## Ethics Statement

This study involved human participants and was approved by the ethics committee of the Psychologische Hochschule Berlin, Berlin, Germany (approval no. AZ: EK201921-II). The participants provided their written informed consent to participate in this study.

## Author Contributions

TW, FB-H, NS, SB, MS, and SG-N designed the study and contributed to the interpretation of data and internal revision of the manuscript. TW managed the translation process of the scale, conducted the statistical analysis, interpretation of data, and wrote the first draft of the manuscript. TW, FB-H, NS, and SG-N executed and supervised the acquisition of data. All authors contributed to and have approved the final manuscript.

## Funding

TW and SG-N are (management committee) members of COST action CA18211: DEVoTION: Perinatal Mental Health and Birth-Related Trauma: Maximizing best practice and optimal outcomes.

## Conflict of Interest

The authors declare that the research was conducted in the absence of any commercial or financial relationships that could be construed as a potential conflict of interest.

## Publisher's Note

All claims expressed in this article are solely those of the authors and do not necessarily represent those of their affiliated organizations, or those of the publisher, the editors and the reviewers. Any product that may be evaluated in this article, or claim that may be made by its manufacturer, is not guaranteed or endorsed by the publisher.
